# The Role of Classroom Culture and Psychological Safety in EFL Students' Engagement

**DOI:** 10.3389/fpsyg.2021.760903

**Published:** 2021-09-29

**Authors:** Xiaowei Tu

**Affiliations:** School of Foreign Languages, Zhongyuan University of Technology, Zhengzhou, China

**Keywords:** classroom culture, psychological safety, student engagement, positive psychology, learner psychology variables

## Abstract

Language learning is a complex phenomenon that is the outcome of an interplay of numerous inter/intra-personal variables. Out of these factors, emotions play a critical role in the whole process of learning. Research approves that positive emotions lead to positive outcomes. This is only obtainable in a positive classroom culture where students feel psychologically safe. If so, they actively engage in the classroom activities for a longer period. However, the macro-effect of classroom culture in EFL/ESL contexts has been limitedly explored. Against this shortcoming, the present article provides a brief account of the definition and conceptualization of classroom culture and its impact on two learner psychology variables (i.e., psychological safety, engagement). Moreover, the dimensions and factors influencing these variables are discussed. Finally, the study offers some implications for different stakeholders in EFL/ESL contexts and enumerates a number of research gaps and future directions for future scholars in this line of inquiry.

## Introduction

Education is a complex phenomenon that is by far more than simple scores on tests. It is about knowledge, understanding, and empowering people to move communities and societies forward (Zulfiquar and Zamir, 2015). For an educational program to be successful, we may present a network of factors related to the teacher, learners, materials, and environment. These elements along with linguistic disparities add to the complexity of second/foreign language education. Among numerous interacting factors in L2 education, classroom culture is the core and a macro element due to its power to influence other constituent components and almost all aspects of language learning (Altun, 2013). It is a broad term referring to the common behaviors, value systems, beliefs, unwritten rules, teaching and learning methods, and relationships in a classroom (Cakiroglu et al., 2012). Moreover, a classroom's culture comprises: (1) its physical environment and location, (2) teacher's ability, teaching style, methodology, and personality, (3) students' engagement and participation, (4) instructional materials, and (5) classroom facilities, sitting arrangements, and space in the classroom (Zulfiquar and Zamir, 2015). Language learning best occurs in a positive classroom culture that cares for students' emotions, agency, autonomy, well-being, active participation, and many other learner-psychology factors.

In such a democratic culture which is context-specific in that each classroom has its own culture, learners' academic achievement boosts exponentially as they feel psychologically safe in a classroom that respects their individuality and preferences (Norton, 2008). Hence, the construct of psychological safety has also a critical role in students' language development. It is originally related to organizational change/learning (Edmondson and Lei, 2014). In SLA, it refers to the protection of students/teachers against academic threats to cause positive growth and mental health during instructional interactions (Kaila, 2020). This highlights the criticality of contexts and classroom culture in education, especially L2 learning which is a venue of numerous interrelated, nested, interacting, and dynamic variables that develop optimally in a milieu that is positive and psychologically safe for students and teachers. Research shows that classroom culture and students' psychological safety meaningfully increase engagement in instructional activities (Kaila, 2020). Engagement is a multi-faceted concept rooted in positive psychology (Wang et al., 2021) that concerns students' degree of involvement in the class. It is affected by many internal and external factors (Guilloteaux, 2016). Two of such factors are undoubtedly classroom culture which needs to be democratic and positive and students' high level of psychological safety. Nevertheless, no study, to date, has explored these three pivotal constructs together in EFL contexts. To do so, this article presented a conceptual review of these learner-psychology variables and offers some future directions.

## Background

### The Concept of Classroom Culture

Classroom culture is a macro-construct that includes almost all aspects of an educational setting and stakeholders' behaviors, beliefs, and practices. As put by Zulfiquar and Zamir (2015), classroom culture refers to the physical environment; teacher's ability, teaching style, methodology, and personality; students' engagement; instructional materials; and classroom facilities. It differs from *classroom climate* in that climate concerns the social-ecological context in which learners operate which can influence their attitudes, perceptions, behaviors, moods, performance, self-concept, and well-being (Moos, 1979). In other words, climate is the overall feeling of stakeholders regarding classroom interactions, involvement, and academic experience (Gabryś-Barker, 2016). On the other hand, classroom culture refers to the shared values, rules, traditions, belief patterns, behaviors, and relationships in a class (Cakiroglu et al., 2012). It is broader and may change only over a long period of time (Gruenert, 2008). Hence, climate can be regarded as “the attitude and mood” of stakeholders about a class, while culture is “the values and belief systems” of a class (Gruenert, 2008).

### Factors Influencing Classroom Culture

Several factors can affect a classroom's culture including learner, teacher, classroom, materials, environment, and relationships. Learner-related factors are their demographic factors, perceptions, and images of others, classroom participation, and first language. Factors related to the teacher are background information, teaching knowledge, expertise, and style. Classroom-related factors include seating, size, equipment, and attractiveness. Moreover, the type and quality of materials used in a class can influence its macro culture. Factors related to the environment include its location, appearance, and being peaceful and silent for better concentration. Finally, teacher-student and student-student relationships in the class can considerably influence its culture (Hussein, 2016).

### Characteristics of a Positive Classroom Culture

A positive classroom culture which can produce many positive academic outcomes has a number of features. It is based on respect and belief in learners' ability; the curriculum and syllabus are real-life, negotiated, and based on learners' needs and wants; the responsibility of learning is shared by all; students have voice, agency, and autonomy; the teacher is the role model, facilitator, feedback-provider, and supporter of students; and the classroom environment is peaceful and democratic helping students and teachers perform their best (Hussein, 2016).

### The Notion of Psychological Safety

The concept of psychological safety has originally been proposed by Kahn (1990) and Schein (1993) for workplace culture and organizational learning. Later, it was popularized and defined by Edmondson and Lei (2014) as a common belief among members of a team/community that it is safe for group members to take interpersonal risks. It concerns the removal of fear from human interactions and bases them on respect and permission (Clark, 2020). In such a context, people feel included, belonged, and safe to learn, contribute, and challenge the current condition without fear (Clark, 2020). Therefore, it plays a significant role in workplace effectiveness, engagement, innovation, cohesiveness, and cooperation (Newman et al., 2017).

The concept is new to SLA referring to the safety that students and teachers feel in the classroom context for taking initiative, interact, and speak out their ideas without being embarrassed, humiliated, and punished. Owing to the complex dynamics of L2 learning, a classroom culture that entails and generates psychological safety for students produces more outstanding and positive outcomes such as learning, cooperation, engagement, motivation, and improved interpersonal communication skills among students. This construct has a macro-effect on EFL/ESL students' academic performance and engagement. It is similar to constructs like academic resilience, immunity, and buoyancy which concern students' and teachers' protections against threats and conflicts in academia. In sum, the concept of psychological safety is the by-product of positive, democratic classroom culture and school climate which are responsible for its establishment, maintenance, and development.

### Student's Engagement: Conceptualizations and Dimensions

**Figure 1:**
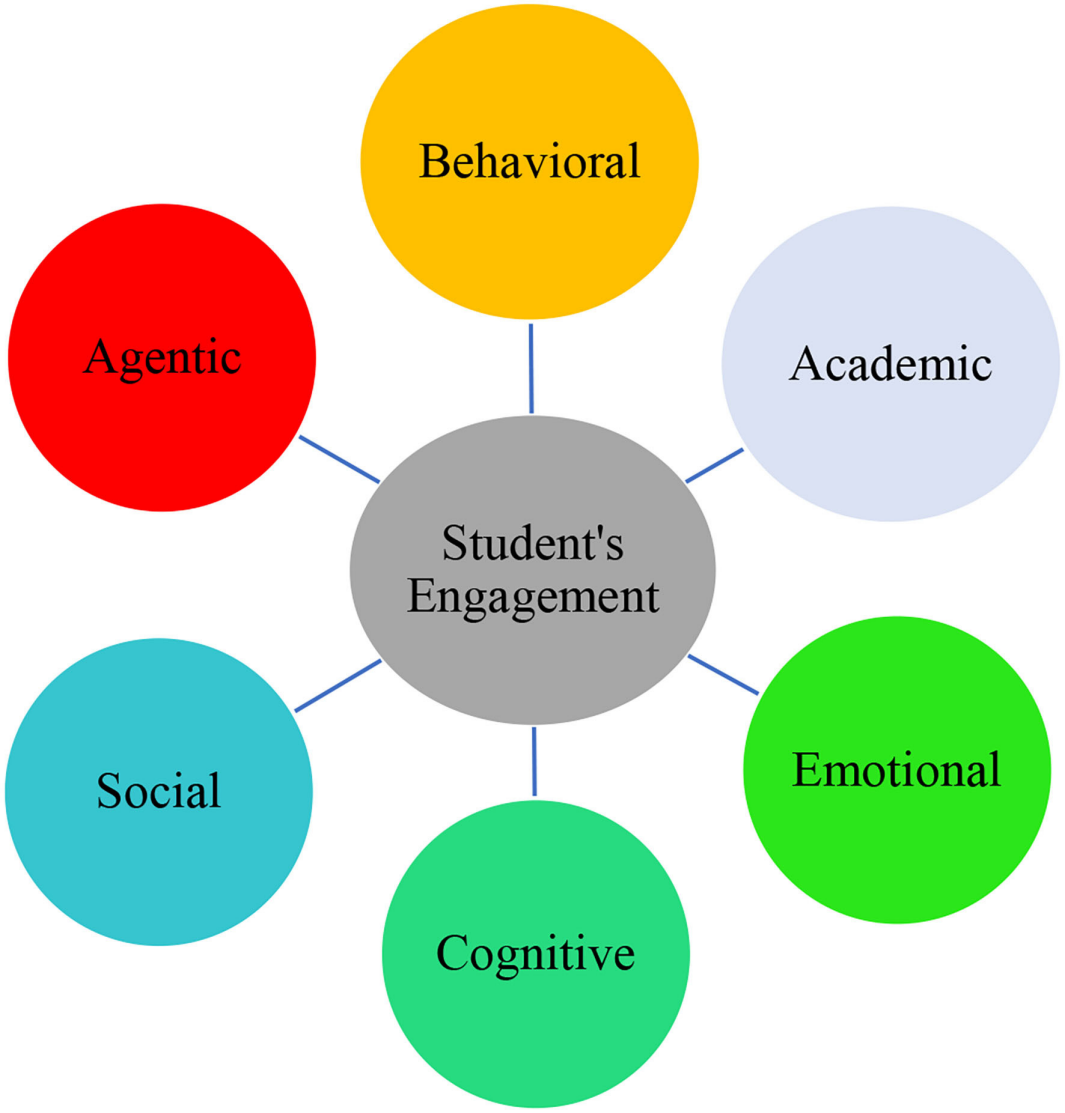
The dimensions of student's engagement.

Student engagement is a multi-faceted construct that is a critical issue in academia throughout the world (Kraft and Dougherty, 2013). It has recently come into vogue by positive psychology researchers who focus on the role of positive emotions in education to their positive outcomes (MacIntyre et al., 2019; Wang et al., 2021). According to Skinner and Pitzer (2012), engagement refers to the degree and quality of students' involvement in classroom activities. It is a direct reflection of intrinsic motivation in students (Elliott and Tudge, 2012). Different researchers have had different conceptualizations of this concept; however, they unanimously confirm that engagement is multi-dimensional and a meta-construct that develops through time and in a positive environment. The dimensions of engagement include behavioral, emotional, cognitive, agentic, academic, and social as illustrated in Figure 1 (Oga-Baldwin, 2019).

By definition, *behavioral engagement* refers to students' obedience and active involvement in the activities by paying attention, participating in the class, involving in tasks, and doing assignment, while *emotional or affective engagement* is about students' inner states and affective reactions in learning such as anxiety, stress, interest, enjoyment, fun, and happiness and so on. Moreover, *cognitive engagement* points to learners' psychological investment in learning and employing complex learning strategies when doing a task. *Agentic engagement* has do to with learners' contribution to the improvement of learning and teaching. Likewise, *academic engagement* refers to one's psychological and behavioral attempts to master academic knowledge and skills (Fredricks et al., 2004). Lastly, *social engagement* concerns students' involvement in a set of classroom tasks that are aimed at arousing students' social interaction and problem-solving (DeVito, 2016).

All of these components are dynamic, teachable, and promotable by teachers' positive instructional practices and a positive, democratic classroom culture that secures students' psychological safety (Quin, 2017). Research shows that students' engagement has different positive academic outcomes (Eccles, 2016). It is correlated with students' achievement, motivation, interpersonal skills, psychosocial adjustment, effective learning, and success (Chase et al., 2015; Jang et al., 2016; Xie and Derakhshan, 2021).

### Factors Affecting Students' Engagement

Like many other dynamic and multi-faceted variables in SLA, engagement is affected by several factors (Collins, 2014). The factors can be divided into *phenomenological, individual-demographic*, and *instructional* factors. Phenomenological factors include task difficulty, ability, culture, task type, and task value, while individual-demographic factors comprise age, gender, and academic grade. Finally, instructional factors refer to teachers' actions, behaviors, motivation to teach, ability, and instructional style. What is missing among these factors is the impact of classroom culture as a macro-factor that can influence each and every aspect of education. Undoubtedly, this reflects the importance of environmental factors as well. As engagement is dynamic, there also exist other variables which can affect it such as personality traits, time, motivation, orientation, attitude, self-esteem, self-efficacy, confidence, interest, and other positive emotion variables.

## Implications, Research Gaps, and Future Directions

In this review article, it was pinpointed that second/foreign language learning does not happen in a vacuum but via a network of interacting variables. This dynamic and nested system has made language teaching and learning a complex phenomenon. For learning to occur, there must exist a positive, friendly, democratic, and supportive instructional environment for learners to feel psychologically safe in the face of adversities and perform best in academia. This is only achievable through a classroom culture that cares for and respects students' emotions and inner states. Therefore, this line of research can have precious implications for EFL/ESL students, teachers, teacher-trainers, materials developers, school principals, and L2 researchers. Considering students, this study can be helpful in that they can become aware of the role of classroom culture in education which is the outcome of many factors including students' actions and rapport with the teacher. Definitely, when they form a friendly rapport with their teachers and peers a positive environment is established that facilitates learning. Similarly, teachers can use the ideas in this mini review to use appropriate techniques, methods, and tools in the classroom which foster the creation of a democratic atmosphere in which their students feel psychologically safe and actively engage in the classroom activities.

Moreover, this study is beneficial for teacher educators in that they can run training courses in which they teach novice teachers macro issues such as how to establish a positive classroom culture and actively involve learners. They can offer proper techniques and strategies which foster the achievement of these objectives. Furthermore, materials developers can utilize this study to develop tasks and activities which promote EFL/ESL students' degree of engagement and psychological safety. School principals can also benefit from this review in that they can help teachers and students in creating a caring and positive culture in the classroom and the school by providing support and required facilities. Finally, L2 researchers can use the propositions made in this research and conduct similar studies on related concepts and in different contexts. In this domain, most of the studies have focused on learner or teacher-related positive emotions and variables and constructs at the macro level have been overlooked. Hence, one area for future research can be studying factors related to school, policy, and planning.

## Conclusion

Similar studies can be done on the correlation of classroom culture/climate and other interpersonal variables (e.g., resilience, mindfulness, care, clarity, rapport, immediacy, self-efficacy, etc.). Qualitative studies are also recommended on EFL students' psychological safety to unpack their views on the factors, antecedents, and outcomes of this variable in their education. Additionally, culture as a mega-construct that affects everything can be examined in this area by running cross-cultural studies on classroom culture and psychological safety. Another gap that needs to fill is that most studies on intra-psychic factors are about learners and teachers' views are less explored. Hence, studying teachers' perceptions and perspectives about the role of classroom culture in SLA is suggested. Furthermore, the mediating role of demographic factors and time in this strand has caught inadequate attention. Consequently, running longitudinal studies in light of demographic variables can add fresh insights about the developmental trajectories of the three variables discussed in this article. These backdrops confirm that this area is still fresh and needs more research.

## Author Contributions

XT drafted the manuscript and submitted to Frontiers in Psychology.

## Funding

This paper was a phased achievement of Research Project funding of the Humanities and Social Sciences Research Project of Henan Universities (Project Title: Study on Female Images in the works of Russian Chinese Writers of Paris in the 20th Century, Project Grant No: 2021-ZZJH-294).

## Conflict of Interest

The author declares that the research was conducted in the absence of any commercial or financial relationships that could be construed as a potential conflict of interest.

## Publisher's Note

All claims expressed in this article are solely those of the authors and do not necessarily represent those of their affiliated organizations, or those of the publisher, the editors and the reviewers. Any product that may be evaluated in this article, or claim that may be made by its manufacturer, is not guaranteed or endorsed by the publisher.
